# The Netrin-1 receptor DCC is a regulator of maladaptive responses to chronic morphine administration

**DOI:** 10.1186/1471-2164-15-345

**Published:** 2014-05-08

**Authors:** De-Yong Liang, Ming Zheng, Yuan Sun, Peyman Sahbaie, Sarah A Low, Gary Peltz, Grégory Scherrer, Cecilia Flores, J David Clark

**Affiliations:** Anesthesiology Service, Veterans Affairs Palo Alto Health Care System, 3801 Miranda Avenue, Palo Alto, USA; Department of Anesthesiology, Perioperative and Pain Medicine, Stanford University School of Medicine, Stanford, USA; Stanford Institute for Neuro-Innovation and Translational Neurosciences, Stanford, USA; Department of Psychiatry, McGill University, Montreal, Canada

**Keywords:** Genetics, Mapping, Opioid, Pain, Addiction

## Abstract

**Background:**

Opioids are the cornerstone of treatment for moderate to severe pain, but chronic use leads to maladaptations that include: tolerance, dependence and opioid-induced hyperalgesia (OIH). These responses limit the utility of opioids, as well as our ability to control chronic pain. Despite decades of research, we have no therapies or proven strategies to overcome this problem. However, murine haplotype based computational genetic mapping and a SNP data base generated from analysis of whole-genome sequence data (whole-genome HBCGM), provides a hypothesis-free method for discovering novel genes affecting opioid maladaptive responses.

**Results:**

Whole genome-HBCGM was used to analyze phenotypic data on morphine-induced tolerance, dependence and hyperalgesia obtained from 23 inbred strains. The robustness of the genetic mapping results was analyzed using strain subsets. In addition, the results of analyzing all of the opioid-related traits together were examined. To characterize the functional role of the leading candidate gene, we analyzed transgenic animals, mRNA and protein expression in behaviorally divergent mouse strains, and immunohistochemistry in spinal cord tissue. Our mapping procedure identified the allelic pattern within the netrin-1 receptor gene (*Dcc*) as most robustly associated with OIH, and it was also strongly associated with the combination of the other maladaptive opioid traits analyzed. Adult mice heterozygous for the *Dcc* gene had significantly less tendency to develop OIH, become tolerant or show evidence of dependence after chronic exposure to morphine. The difference in opiate responses was shown not to be due to basal or morphine-stimulated differences in the level of *Dcc* expression in spinal cord tissue, and was not associated with nociceptive neurochemical or anatomical alterations in the spinal cord or dorsal root ganglia in adult animals.

**Conclusions:**

Whole-genome HBCGM is a powerful tool for identifying genes affecting biomedical traits such as opioid maladaptations. We demonstrate that *Dcc* affects tolerance, dependence and OIH after chronic opioid exposure, though not through simple differences in expression in the adult spinal cord.

**Electronic supplementary material:**

The online version of this article (doi: 10.1186/1471-2164-15-345) contains supplementary material, which is available to authorized users.

## Background

Opioids are the drugs that are most commonly used for the treatment of chronic pain. Unfortunately, maladaptive responses to opioids (tolerance, dependence and OIH (opioid-induced hyperalgesia)) have greatly limited their clinical utility [1, 2]. As examples, the gradual loss of analgesic effect, worsening pain despite dosage increases, and greatly exaggerated postoperative pain have all been attributed to OIH [3–5]. Despite decades of study, we have no specific therapies that can limit or overcome the problems associated with chronic opioid use. The lack of new approaches results from our limited understanding of the mechanisms mediating the maladaptive responses.

Genetic analysis offers a hypothesis-free approach that could be used to discover the mechanisms mediating maladaptive responses to opiates, and this information could lead to novel therapeutic strategies. Inter-individual genetic differences among human populations have been shown to explain a substantial fraction of the variance in clinically relevant responses to opioids [6, 7]. We expect genetic approaches in mice to also be informative, since there are very large differences in the response of inbred strains to opiates, including the development of opioid analgesia, tolerance, dependence and hyperalgesia [8–14]. In fact, HBCGM, which used SNP databases generated from limited sequence information, has identified several involved genes including those coding for β2-adrenergic and 5-HT3 receptors that affect opiate responses, and these findings were translated to humans [8, 15].

Mouse geneticists have classically used one of two approaches to analyze a murine genetic model. The first approach is to conduct linkage (quantitative trait loci, QTL) studies on mice derived from the interbreeding two or more strains of mice. The identification of the *Gnao1* as a regulator of opioid physical dependence provides an important successful example for pain research [16]. The recombinant inbred strains produced by the Collaborative Cross have enhanced the power of this approach [17, 18], and sophisticated data analysis tools are now available [19]. As an alternative approach, HBCGM can be used to conduct a genetic association study, which correlates phenotypic responses with the patterns of genetic variation present in a panel of 10 or more inbred strains [20]. The pattern of genetic variation is represented by haplotypes, which were constructed from analysis of sequence data obtained from a large number of inbred strains. This approach has identified several genes involved in opioid maladaptive responses [12–14]. However, the lack of genome-wide sequence information for an adequate numbers of inbred mouse strains has previously limited the power of this technique, as have limitations in the tools that are required to analyze the large amount of sequence information. To overcome these limitations, we have developed a HBCGM method that uses a whole-genome SNP database that was generated by analysis of whole genome sequence data for 25 inbred strains^a^. Here, we use this new tool to address the public health problems associated with chronic opioid use, and identify a novel gene that is involved in maladaptive opioid responses.

## Results

### Opioid-related behavioral responses

**Figure 1:**
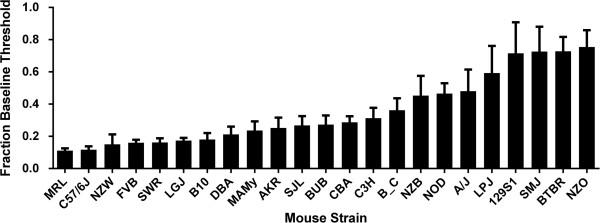
**Opioid-induced hyperalgesia in 23 strains of inbred mice.** For each strain, mice were tested for mechanical nociceptive thresholds at baseline and after 4 days of morphine treatment. Nociceptive thresholds after morphine treatment were divided by baseline values to obtain the fraction of baseline values. For each strain n = 8 mice. Mean values are displayed +/- S.E.M.

The phenotypic data for opioid responses including analgesia, tolerance, dependence and OIH was collected for 23 inbred strains of mice. Subsets of this data were used in previous mapping efforts [8, 13, 14]. The behavior response of principal interest in these studies was OIH; a mechanical stimulus was used to characterize changes in nociceptive thresholds at baseline and after chronic morphine treatment (Figure 1). The percent reduction of mechanical nociceptive thresholds after chronic opioid treatment varied approximately 7-fold across the 23 strains analyzed. Of note, there was not a significant correlation between baseline mechanical threshold and the degree of OIH observed after chronic morphine treatment (P > 0.05). Additional file 1: Figure S1 provides data for other chronic opioid adaptive traits analyzed here, including: hindpaw OIH measured using Hargreave’s thermal testing, OIH measured using tail-flick thermal testing, analgesic tolerance and physical dependence.

Although we focus on identifying genetic factors affecting OIH, we previously noted (using data obtained from a smaller number of strains) that the strain dependencies of OIH, tolerance and physical dependence were correlated [13]. Furthermore, the genes identified by analysis of one opioid adaptive trait were found to affect other adaptive traits when subsequently tested using pharmacologic agents and transgenic animals [8, 12, 13]. We therefore analyzed the correlations between the opioid adaptive traits, and found moderate levels of correlation between the index trait (mechanical OIH) and several opioid adaptive phenotypes (Table 1). After correction for false discovery, however, only the correlation between mechanical and thermal OIH remained significant. Strains displaying more OIH assessed using a mechanical stimulus tended to show morphine-induced hyperalgesia to thermal stimuli as well. Moreover, stains developing more hyperalgesia also tended to show more tolerance and physical dependence on morphine. These correlations are consistent with the hypothesis that some of the same genetic variants affect diverse opioid adaptive traits.

**Table 1:** **Correlations between mapped traits**

	OIH Mech	OIH TF	OIH Therm	Tolerance	Dependence
**OIH Mech**	1	0.46*	0.58†	-0.50*	-0.21
**OIH TF**		1	0.43*	-0.18	0.24
**OIH Therm**			1	-0.38	0.11
**Tolerance**				1	0.41*
**Dependence**					1

Strain-specific trait data related to the chronic administration of morphine was subjected to Pearson correlation analysis. The resulting correlation coefficients are displayed. P values were adjusted using the Benjamini-Hochberg method to control for false-discovery rate [56]. ^†^: adjusted P value < 0.05 *: raw p value < 0.05.

### HBCGM results

**Figure 2:**
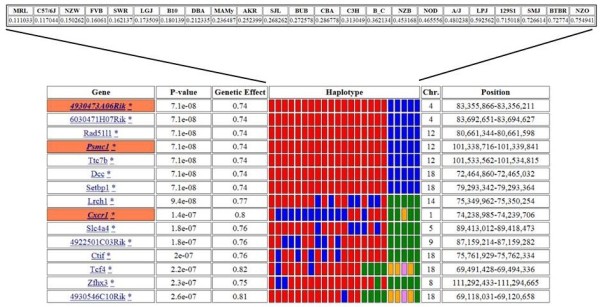
**Whole-genome computational genetic mapping.** The strain-specific mechanical OIH data was used for whole genome halplotypic mapping. The top panel provides the input data for the 23 strains used. The bottom panel provides the name of the genes to which the mapped haplotype blocks belong, the p-value for association of the block with the trait data, the maximum genetic effect attributable to the mapped block, and the color-coded haplotypes for each of the 23 strains. The three highlighted genes contain SNPs predicted to change amino acid sequence. In addition, chromosomal location of the mapped blocks is provided.

Whole-genome HBCGM was used to analyze the opioid-related traits. We focused on mechanical OIH as the trait of primary interest, and examined the 15 genes whose haplotypic pattern were most strongly associated with this response (Figure 2). The 7 genes that were most strongly associated with this phenotype had haplotype blocks that partitioned the strains in an identical fashion, and therefore generated the same p value. None of the top 15 genes had previously been associated with OIH, as determined by a search of the PubMed database (using the gene name and “opioid-induced hyperalgesia” or “opioid” or “morphine.”) Although these 15 genes all had highly significant statistical correlations with mechanical OIH, it was difficult to determine which was most likely to be a causative genetic factor. Therefore, we assessed the robustness of the mapping results as described in Methods. In brief, we determined which of the mapped genes was most resistant (i.e. robust) to a change in the number of strains used for the genetic mapping. Of note, 13 of the 15 genes that were most robustly associated with OIH, were the genes that were most strongly associated with the initial analysis of the OIH response (Table 2). However, the robustness analysis significantly altered the gene order. The deleted in colorectal carcinoma *(Dcc)* gene was the highest ranked gene by the robustness analysis. Studies have linked this gene to behavioral effects of acute and repeated exposure to stimulant drugs of abuse, cognitive flexibility, learning and memory, and movement disorders [21–25].

**Table 2:** **The ranking of mapped mechanical OIH genes using robustness scoring**

Gene	Score
*Dcc*	395.65
*X4930473A06Rik*	392.33
*Setbp1*	377.11
*Rad51l1*	375.21
*X6030471H07Rik*	374.88
*Psmc1*	374.67
*ttc7b*	374.53
*X4930546C10Rik*	368.11
*Slc4a4*	362.14
*Ctif*	353.74
*Cxcr1*	343.80
*Lrch1*	342.93
*Tcf4*	340.13
*Rora*	337.48
*Vil1*	335.24

Genes mapped to the trait of mechanical OIH were ranked after subjecting the data to the robustness scoring procedure described in Methods. Higher scores indicate more robust mapping results.

The *Dcc* gene is large and extends over 1.09 MB of chromosome 18. The highly correlated haplotype block is near the 5’ end of the first intron. The *Dcc* gene encodes a receptor for the axonal guidance cue netrin-1, which is best described for its role in the organization of neuronal connectivity during development, but it has also been shown to play a role in behavioral and synaptic plasticity in the adult brain [21, 25–27]. DCC-mediated netrin-1 signaling is also implicated in cell migration, cell death, and angiogenesis [19, 26–29]. Two SNPs in this gene induce amino acid changes. Further analysis revealed that the variation pattern of these two amino acid changing SNPs divided the 23 strains into 3 groups with low (<0.5), low and high (>0.5) fractional changes in baseline mechanical nociceptive thresholds after administration of morphine (Additional file 2: Figure S2). All three strains with the A713V amino acid substitution were highly resistant to OIH measured using both thermal and mechanical stimuli, and showed little development of tolerance as well.

To identify genes that could potentially affect other opioid adaptive traits, we also analyzed the number of times that a gene had a haplotype that was associated with any one of the opioid-response traits analyzed; this analysis also evaluated the strength of the association. Of the associated genes, *Dcc* was the gene that was most strongly associated with this group of opioid adaptive phenotypes (Table 3). Overall, five genes appeared in the top 15 genes identified using both the robustness and combinatorial analysis: *Dcc*, *Rora*, *Tcf4*, *Ctif* and *Lrch1*.

**Table 3:** **The integrated ranking of genes associated with the opioid adaptive traits**

Gene	Score
*Dcc*	23.55
*Sgpp2*	22.51
*Sgcz*	21.51
*Gm16724*	21.06
*Kank1*	20.95
*Rora*	20.73
*Myo5b*	20.53
*Tcf4*	20.20
*Dpp10*	20.07
*Ctif*	20.01
*Lhfpl3*	20.00
*Pik3c2g*	19.95
*Erbb4*	19.92
*L3mbtl4*	19.68
*Lrch1*	19.59

The genes most strongly associated with the five opioid adaptive phenotypes under study considered together are listed in the table. These traits include: mechanical OIH (Mech OIH), OIH measured using tail flick data (TF OIH), OIH measured using paw withdrawal testing (Therm OIH), morphine tolerance (Tolerance) and physical dependence (Dependence). Higher scores indicate stronger combined associations with the group of five traits.

### Altered Dcc expression affects opioid adaptations but not nociceptive neurochemistry

**Figure 3:**
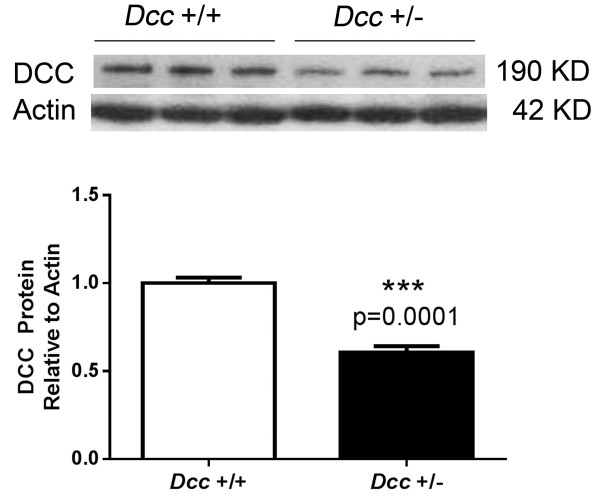
**Spinal cord protein levels of DCC in wild type and heterozygous mice.** Lumbar spinal cord tissue from wild type and heterozygous *Dcc* mice were analyzed using Western analysis. The quantification of the DCC specific band in the immunoblots is shown. For these experiments n = 5 mice per genotype. Data are displayed as mean values +/- S.E.M., ***p < 0.001.

Given the genetic mapping results, we compared the responses of mice that were heterozygous for the *Dcc* gene (on a C57BL/6 background) with those of their wild type (C57BL/6 J) littermates after chronic morphine treatment. Adult heterozygous *Dcc* mice were used because homozygous *Dcc* knockout mice die within a few hours of birth [30]. To determine whether adult *D*cc heterozygous mice have reduced DCC protein levels we conducted immunoblot analysis of spinal cord homogenates and found that the *Dcc* heterozygous mice had ~50% lower DCC protein levels than wild-type littermates (Figure 3).

**Figure 4:**
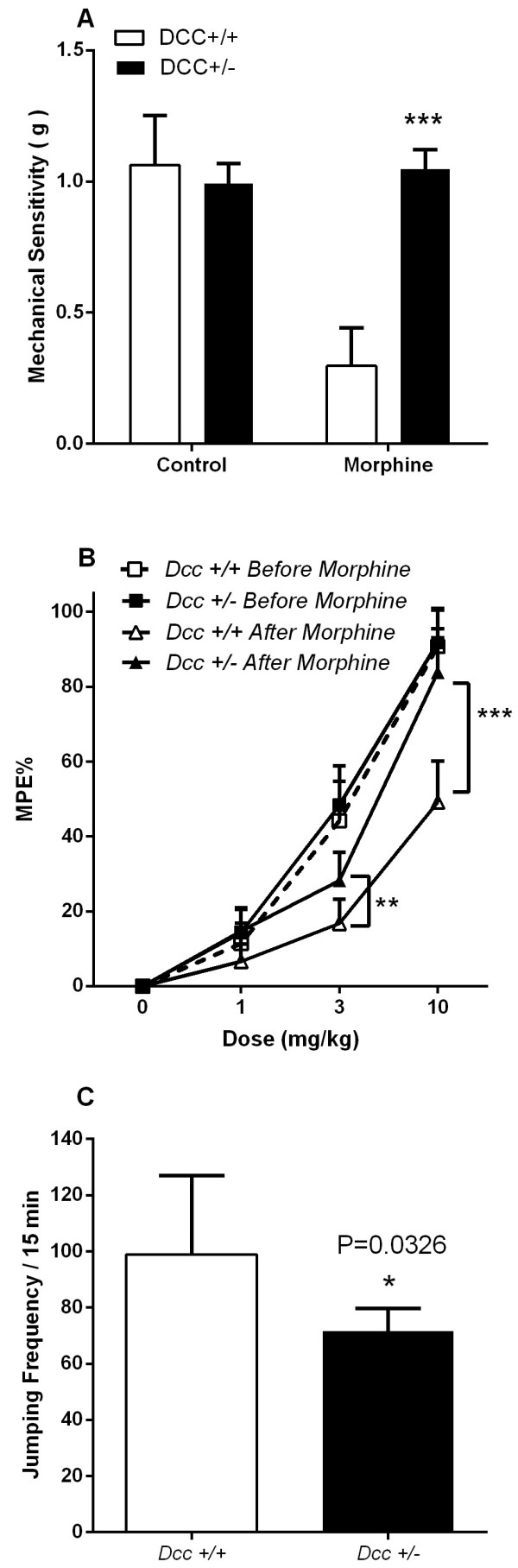
**The effects of chronic morphine treatment on** ***Dcc*** **wild-type and heterozygous mice.** In these experiments littermate *Dcc* wild-type (C57BL/6 J) and *Dcc* heterozygous mice were tested using the same chronic morphine paradigm used for the inbred strain mapping experiments. Panel **A**: Mechanical OIH measurements; Panel **B**: Morphine dose–response relationships before and after chronic morphine treatment; Panel **C**: Physical dependence measured using naloxone-precipitated jumping behavior. For these experiments n = 11 mice per genotype. Data are displayed as mean values +/- S.E.M., *p < 0.05, **p < 0.01, ***p < 0.001.

Despite the difference in DCC protein expression in the spinal cord, *Dcc* heterozygous and wild type mice did not differ in their basal (prior to morphine treatment) nociceptive responses to thermal and mechanical stimuli, nor in the extent of opioid analgesia observed after acute administration of morphine to drug naïve mice (Figure 4). In contrast, wild-type mice displayed markedly reduced threshold levels of mechanical hypersensitivity after chronic opioid treatment (i.e. OIH), while *Dcc* heterozygous mice did not develop OIH. In addition, *Dcc* heterozygous mice had substantially reduced tolerance to morphine after several days of treatment and developed moderately less physical dependence on morphine than their wild-type littermates (Figure 4).

**Figure 5:**
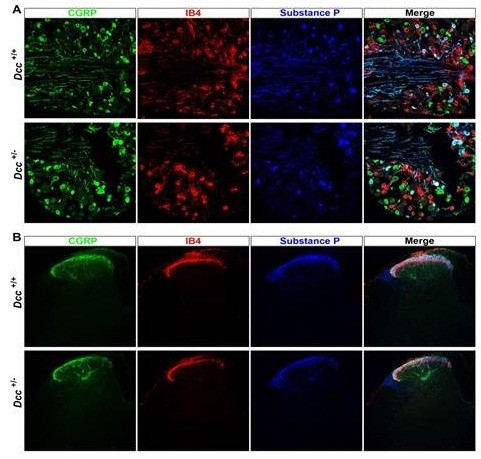
**Intact distribution and central projections of peptidergic and non-peptidergic nociceptors in** ***Dcc*** **heterozygous mice.** Panel **A**: Triple immunolabeling experiment showing similar distribution of CGRP-immunoreactivity, substance P-immunoreactivity, and binding of isolectin B4 (IB4) in dorsal root ganglion sections from *Dcc* wildtype and heterozygous littermates. Panel **B**: Triple immunolabeling experiment indicating that in the spinal cord dorsal horn the typical laminar organization of central terminals of peptidergic (CGRP- and substance P-expressing) and non-peptidergic (IB4-binding) nociceptors is intact in *Dcc* heterozygous mice, compared to wildtype littermates.

To begin to discern the mechanisms underlying this differential response to chronic morphine in *Dcc* heterozygous mice, we examined the structural and neurochemical architecture of the dorsal horns of the spinal cords and of dorsal root ganglia (DRG) of wild-type and *Dcc* heterozygous mice. We analyzed the spinal cords and DRG for peptidergic (CGRP, SP) nerve terminals projecting to lamina I and outer lamina II, as well as non-peptidergic (IB4) nerve terminals projecting to the dorsal border of inner lamina II; and for the levels of DRG expression of selected markers. The normal architecture of these sensory endings in the dorsal spinal cords is preserved in the *Dcc* heterozygous mice, and the expression of the DRG markers in *Dcc* heterozygous mice is also unchanged (Figure 5). Thus, the changes in opioid responses observed in *Dcc* heterozygous mice do not appear to be caused by a gross alteration in spinal cord or DRG architecture.

### Treatment with morphine does not change Dcc (gene and protein) expression in spinal cord tissue

**Figure 6:**
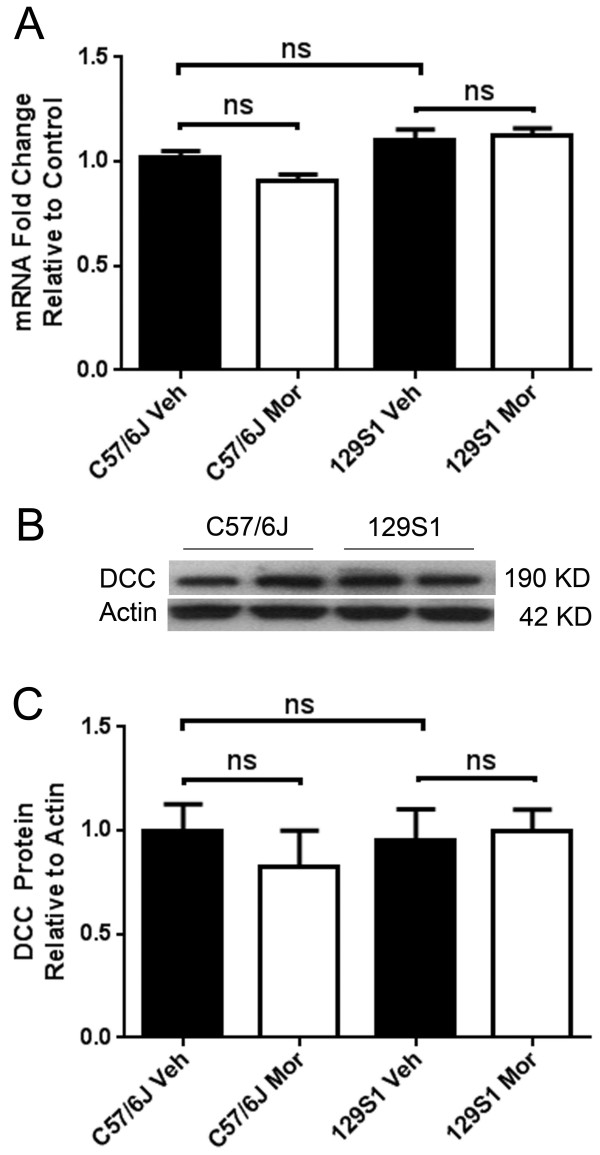
**Expression of** ***Dcc*** **in spinal cord tissue after chronic morphine treatment in C57/6 J and 129S1 mice.** Panel **A**: The mRNA levels of *Dcc* were unchanged after chronic morphine treatment in both C57/6 J and 129S1 mice. Values are displayed as the mean ± SEM, n = 6. ns, no significant difference. Panel **B**: Western blot assay demonstrated that there was no significant difference in baseline levels of DCC in spinal cord tissue from C57/6 J and 129S1 mice. Panel **C**: The spinal cord protein levels of DCC were unchanged after chronic morphine treatment in both C57/6 J and 129S1 mice. Values are displayed as the mean ± S.E.M., n = 4. ns, no significant difference. Veh = vehicle, Mor = morphine treatment.

Neuroplastic changes within the spinal cord have been associated with the development of OIH and opioid tolerance, and many genes undergo changes in spinal cord gene expression after chronic morphine exposure [31]. Furthermore, DCC protein levels in the CNS were observed to be altered after repeated exposure to amphetamines [32]. Therefore, we hypothesized that morphine alters *Dcc* expression in mouse spinal cord tissue after repeated exposure. However, the levels of *Dcc* expression in the spinal cord of C57BL/6 J mice (a morphine adaptive strain) and in a very adaptation-resistant strain (129S1) were unchanged after morphine treatment measured at both the mRNA and protein levels (Figure 6). In addition, the DCC protein levels in spinal cord tissue in these two strains were indistinguishable under control conditions.

## Discussion

Our experiments were designed to help us better understand the maladaptations to chronic opioid administration that limit the clinical utility of our most powerful class of analgesics. A significant number of molecules have been mechanistically linked to opioid maladaptations including cell surface receptors, ion channels, drug transporters and other proteins. However, very few studies have leveraged genetic techniques to discover the genes involved in hypothesis-free fashion. One technique, HBCGM, has shown great promise in this respect, but has to this time been limited by the lack of whole genome sequence information and the availability of the informatics required to use this information. The present results demonstrate that robust mapping studies can be completed using newly developed tools and relatively easily obtainable whole genome sequence information thus largely overcoming the previous limitations. Our principal findings were, 1) that multiple candidate genes could be identified by the whole genome mapping of 23 strains of common inbred mice, 2) that techniques such as combining the mapping results of related phenotypes and re-sampling of strain information can be used to help rank the candidate genes for in-depth structural and functional analysis, 3) that the *Dcc* gene is computationally associated with several opioid maladaptations of interest, and 4) that *Dcc* heterozygous mice display resilience to the development of opioid maladaptations. Thus we have used a refined set of genetic mapping tools to discover and subsequently confirm a novel gene controlling maladaptations to chronic opioid use.

The *Dcc* gene was discovered as investigators searched for causes of colorectal carcinoma [33]. This gene is deleted in approximately 70% of colorectal carcinomas leading to the hypothesis that *Dcc* is a tumor suppressor gene [28, 34]. At the same time a seemingly very separate type of function has been demonstrated for DCC protein, that of an axonal guidance molecule serving as a receptor for netrin-1 [26, 27]. In addition to misrouting of axons caused by DCC deficiency, reduced levels of this protein alter patterns of synaptic connections within the developing CNS as well as synaptic plasticity within the adult brain [21, 23, 35]. The integrity of DCC-mediated netrin-1 signaling is required in the development of multiple central and peripheral nervous system structures including the prefrontal cortex, visual system and spinal cord [23, 36–38]. Major structural and organizational changes such as failure of axons to cross the midline have been attributed to DCC signaling insufficiency, even when loss of *Dcc* expression and function are only partial [21, 24, 39]. The *Dcc* gene itself codes for a single transmembrane spanning receptor protein. It controls the guidance of axons (and dendrites) towards sources of netrin-1, but it is also involved in additional events including axon differentiation and synaptogenesis [21, 27, 35, 37]. Our observations of spinal cord nociceptive fiber innervation suggest that certain basic nociceptive connections may be intact in *dcc* heterozygous mice, but we cannot rule out functionally important spinal cord structural differences that would require more detailed study to detect.

While *Dcc* has not to this point been reported as a specific gene regulating pain or opioid-related phenotypes, the *Dcc* gene has received significant attention in addiction-related research. Much of this work has centered on the development and adult plasticity of the mesocorticolimbic dopamine system, a key neural substrate of the actions of drugs of abuse [40, 41]. In adult rodents, sensitizing regimens of amphetamine lead to up-regulation of DCC protein levels in the ventral tegmental area (VTA), the cell body regions of mesocorticolimbic dopamine neurons. Amphetamine-induced increases in VTA DCC protein levels are prevented by co-administration of NMDA receptor antagonists [32], seems to lead to changes in synaptic plasticity, and is required for the development of behavioral sensitization to stimulants [25]. Notably, adult *Dcc* heterozygous mice do not develop behavioral sensitization to amphetamine and fail to show reorganization of VTA synaptic connectivity [25]. Similarly, exposing adult *Dcc* heterozygous mice to morphine resulted in a reduced severity of opioid maladaptations. It is possible that the reduction in morphine-induced effects in the heterozygous mice is the result of impaired morphine-induced spinal synaptic plasticity especially in light of DCC’s link to the NMDA receptor, a key structure in OIH and tolerance [3].

In our studies using adult mice we identified strong effects of DCC on three clinically important opioid maladaptations. Since none of these would be considered directly analogous to the stimulant-related phenotypes studied by other investigators, DCC would not likely have been targeted for study in relation to the effects of opioids based on the existing stimulant drug response data alone. Rather, the genetic analysis formed the basis for our hypotheses. Distinct from the results obtained using stimulant drugs, we failed to identify morphine-induced effects on *Dcc* expression either at the mRNA or protein levels in spinal cord tissue, a major neuronal relay station involved in opioid tolerance and opioid-induced hyperalgesia [3, 42]. However, such drug-induced changes may not be necessary if functionally important but as yet unidentified differences in CNS structure between the strains of mice exist even before drug exposure, or if different *Dcc* alleles cause differences in function rather than expression. The latter possibility might be caused by one of the identified SNP variants predicted to change amino acid sequence. In this respect the A713V variant is of great interest as it appears to be associated with a high versus low likelihood of developing either OIH or opioid analgesic tolerance. Though the alanine to valine substitution involves two similar hydrophobic amino acids, similar changes have been shown to cause profound changes in glycine receptor function [43]. Importantly, baseline nociceptive thresholds and analgesic potency of morphine were unaltered in heterozygous mice suggesting that reduced DCC availability may still be sufficient to support the basic development and maintenance of primary pain-related circuitry, but that the ability of adult animals to adapt to sustained morphine administration is reduced. For example, changes in the formation of synapses within the spinal cord dorsal horn or alterations in the density of descending modulatory fibers from the medulla, another CNS center strongly associated with opioid adaptations [44, 45], may modulate the *Dcc* genetic effects. Alternatively, DCC is known to be involved in formation of the locus coeruleus [46], a major brain center linked to descending noradrenergic control of spinal pain signaling and opioid tolerance [47]. Focused studies addressing these hypotheses could be designed.

## Conclusions

In this report we provide one of the first examples of the use of whole-genome murine HBCGM to successfully map a drug-related behavioral trait. We have demonstrated the utility of testing the robustness of the mapping results by the re-analysis of strain subgroups and by combining the results of mapping of related phenotypes. While we focused on the function of our top ranked gene, *Dcc*, many additional gene variants are likely important. Given the design of the tools we have developed, further increasing their power by sequencing additional strains and measuring additional phenotypes of interest should be straightforward. Though the collection of phenotypic data from large numbers of inbred strains of mice can be an arduous process, especially for complex behavioral paradigms, such efforts may pay large dividends in the form of novel hypotheses generated from whole genome analysis.

## Methods

### Animal care and use

All experimental protocols were reviewed and approved by Veterans Affairs Palo Alto Healthcare System Institutional Animal Care and Use Committee prior to beginning the work. All mice used in this series of experiments were kept under pathogen-free conditions, 4 mice per cage with a 12 h light/dark cycle and an ambient temperature of 22 ± 1°C. Food and water were available *ad libitum*.

All mice used for the mapping experiments were male and were obtained from The Jackson Laboratory (Bar Harbor, MA) at 11–12 weeks of age, and were kept in our facility a minimum of 1 week prior to use in experiments. The identity of each of the inbred strains used and the Jackson Laboratories catalog number are listed in Additional file 3: Table S1. Mice heterozygous for the deleted in colorectal carcinoma (*Dcc*) gene and wild-type littermates were bred from animals initially supplied by S. Ackerman (The Jackson Laboratory) [48]. The original *Dcc*^tm1Wbg^ mice [30] have been crossed back to C57BL6/J for a minimum of 36 generations. Cryopreserved material of this mouse strain is available at the Mutant Mouse Regional Resource Centers (https://www.mmrrc.org/catalog/StrainCatalogSearchForm.php?search_query=030626).

### Chronic morphine administration

After baseline nociceptive testing, morphine (Sigma Chemical Co.) was administered to mice subcutaneously (s.c.) 20 mg/kg twice per day on days 1–3 and 40 mg/kg twice per day on day 4 in 50–100 μl volumes of 0.9% NaCl similar to our previous protocols for generating opioid-induced hyperalgesia [12–14].

### Behavioral testing

Thermal withdrawal latency (Hargreaves’ testing) - Response latencies to noxious thermal stimulation of the plantar surface of the hindpaw were measured as described previously [13]. Briefly, mice were placed on a temperature controlled glass platform (29°C) in plastic cylinders. After acclimation, focused light was focused on the plantar surface of the hind paw between the foot pads. The time to withdrawal of the foot was measured to 0.1 sec, and a 20-second cutoff was used to prevent tissue damage. The light beam intensity was adjusted to provide an approximate 10 second baseline latency for the C57BL/6 index strain prior to morphine treatment and the same light intensity was used for all subsequent experiments for all strains. Two measurements were made per animal per test session. Latencies were measured at baseline, and again 18 hours after the final dose of morphine when thermal and mechanical OIH were maximal. Our index of thermal OIH was calculated as the percentage decrease in baseline thermal withdrawal latency resulting from chronic morphine administration.

Mechanical withdrawal thresholds - Mechanical withdrawal testing was performed as described previously [14]. Briefly, mechanical nociceptive thresholds were assayed using nylon von Frey filaments according to the “up-down” algorithm described by Chaplan et al. [49]. In these experiments mice were placed on wire mesh platforms in plastic cylinders. After acclimation, fibers of sequentially increasing stiffness were applied to the plantar surface of a hind paw according to the up-down paradigm. Testing proceeded in this manner until 4 fibers had been applied after the first one causing a withdrawal response allowing the estimation of the mechanical withdrawal threshold using curve fitting of the response data [50]. Our index of mechanical OIH was calculated as the percentage decrease in baseline mechanical nociceptive threshold resulting from chronic morphine administration.

### Morphine dose–response

Cumulative morphine dose–response curves were constructed as described previously [11, 12]. Briefly, the thermal withdrawal latencies of gently restrained mice were measured using a tail-flick apparatus (Columbus Instruments, Columbus, OH) with 0.1 second precision. Two measurements were made per mouse. The lamp intensity was the same for all strains. For the assessment of morphine analgesic tolerance, these dose–response experiments were made on morphine naïve mice and again 18 hours after the final dose of morphine given as part of the chronic morphine administration protocol. The cumulative doses of morphine used were 0,1,2,4,8,16 and 32 mg/kg s.c.. Tail flick latency was determined 25 minutes after morphine administration as demonstrated previously to be the time of peak morphine effect. The parameter %MPE (percent maximal possible effect) was determined according to the following formula:

### Physical dependence

Morphine physical dependence was measured using methods previously reported by our laboratory [11, 12]. Mice were treated with morphine for 4 days according to the standard morphine administration protocol for use in this paradigm. Dependence was assessed 18 hours after the final dose of morphine was given. To precipitate withdrawal, naloxone (Sigma Chemical Co.) 10 mg/kg was injected s.c. in 50 μl 0.9% NaCl. After naloxone administration, mice were placed in the same clear cylinders used for the mechanical nociception assays. The number of jumps made during the following 15 minutes was counted.

### Genetic mapping

Whole-genome computational genetic mapping- Whole-genome variation information was obtained from public data bases (10 inbred strains) [51] and through next-generation sequencing of commercially obtained DNA (The Jackson Laboratory) for the remaining 13 strains (Beijing Genomics Institute, BGI) [52]. Haplotype-based computational genetic mapping (HBCGM) was conducted as described previously [53], and used to identify those genomic regions whose genetic variation pattern was correlated with input phenotypic values for the 23 inbred strains utilized in these studies.

Computational genetic mapping testing for robustness- To assess the robustness of HBCGM using different subsets of input strain data, we developed software that could automatically perform a re-sampling analysis. For each iteration, a number (3, 4, 5 or 6) was randomly selected to determine the number of strains that would be randomly excluded from the 23 total available strains. This number of randomly selected strains was then excluded from mapping analysis. The haplotypes identified with p < 0.01 were tabulated. This process was repeated 100 times. If a re-sampled strain panel was identical to one that had already been re-sampled, this iteration was disregarded, and another subset was randomly chosen using the same procedure. To measure the overall association of a gene with the phenotype among the 100 random re-sampled subsets, we defined a score based on p values obtained, which was calculated as: , where *p*_*i*_ was the resulting p value for that gene in the i^th^ re-sampled experiment (if the p value was bigger than the cutoff and therefore censored in the result, *p*_*i*_ was set to 0.01, i.e. the cutoff). Using this approach, genes with larger scores have stronger overall correlation. The genes were then ranked by their calculated scores. Using a cutoff of 0.001 did not change the rankings of the top 50 genes.

Integration of computational genetic mapping results across related traits- To measure the overall association of a gene with multiple related traits, HBCGM was first applied to evaluate each trait. The number of times any region corresponding to this gene was associated with a trait of interested (p < 0.01) was counted. To take the strength of association into account, a score similar to that defined for robustness test was also calculated: , where *i* runs through all traits of interest and *p*_*i*_ was defined as previously described. In general, genes with multiple highly significant associations have high scores and therefore are of higher interest.

### Protein isolation and analysis

Mice were first euthanized by carbon dioxide asphyxiation and spinal cord tissue was harvested by extrusion. Lumbar spinal cord segments were dissected on a chilled surface. The lumbar area of the spinal cord was readily identified by its enlargement, and the area spanning the approximate L3 through S1 segments was harvested. Dissected tissue was then quick-frozen in liquid nitrogen and stored at -80°C until required for analysis. Spinal cords were dissolved at 4°C in RIPA buffer (50 mM Tris–HCl, pH 8.0, 150 mM sodium chloride, 1.0% NP-40, 0.5% sodium deoxycholate, and 0.1% sodium dodecyl sulfate) in the presence of protease inhibitor cocktail (Roche, Mannheim, Germany). Equal amounts of protein (20 μg) were loaded for SDS-PAGE (10% Tris–HCl acrylamide gel) and electrotransferred onto a polyvinylidene difluorided membranes as described previously [54]. Blots were probed with the primary antibody *of* DCC monocolonal antibody (1:1000, BD Pharmingen, San Diego, CA) at 4°C overnight. β-actin (Sigma Chemical, St. Louis, MO) was used as an internal control. This antibody produces a single band at approximately ~185 kDa on Western blots consistent with the predicted weight of DCC. The specificity of this antibody has been validated previously using embryonic tissues from *Dcc* knockout and *Dcc* wild type embryos [55]. The band intensity was quantified using National Institutes of Health image J analysis software (version 1.44, Bethesda, MD).

### RNA isolation and analysis

The spinal cord samples were dissected and stored as described above. For RNA and real-time quantitative PCR, total RNA was isolated from spinal cord using the RNeasy Mini Kit (Qiagen, Valencia, CA) according to the manufacturer's instructions. The purity and concentration were determined spectrophotometrically. Reverse transcription was accomplished using a First Strand complementary DNA Synthesis Kit (Invitrogen, Carlsbad, CA). Real-time PCR was performed in an ABI prism 7900HT system (Applied Biosystems, Foster City, CA). All PCR experiments were performed using the SYBR Green I master kit (Applied Biosystems). The Dcc primers were purchased from SABiosciences (Catalog number: PPM03679F, SABiosciences, Valencia, CA). The sequences for the GAPDH primers were (forward primer) 5-CTGGAGAAACCTGCCAAGTATGATG-3 and (reverse primer) 5-GAGACAACCTGGTCCTCAGTGTAGC-3. Quantification was accomplished according to the standard curve method. In order to achieve the same PCR efficiency for each analyte, 1:10 serial dilutions of cDNA were used to construct standard curves for *Dcc* and GAPDH. The R2 values for the standard curves for each analyte approached 1.0, suggesting the same amplification efficiency in the PCR reactions under these conditions. Melting curves were performed to document single product formation and agarose electrophoresis confirmed product size. As negative controls, RNA samples that were not reverse transcribed were run. Data were normalized to GAPDH mRNA expression.

### Immunohistochemistry

Mice were deeply anesthetized with 100 mg/kg sodium pentobarbital and perfused transcardially with 10 mL of 0.1 M PBS followed by 30 mL of 3.7% formaldehyde solution (Sigma-Aldrich). The lumbar dorsal root ganglia (DRG) and spinal cord were dissected, post-fixed in the same fixative for 4 hrs, and cryoprotected overnight in 30% sucrose in 0.1 M PBS. Spinal cords were sectioned on a cryostat at 40 μm and processed as free-floating sections. DRG were sectioned at 20 μm and processed on slides (Superfrost, Fisher Scientific). Tissue was first incubated for at least 1 hr in 0.1 M PBS with 0.3% Triton X-100 (Sigma-Aldrich) plus 5% normal donkey serum (NDS, EMD-Millipore) blocking solution. Primary antibodies were diluted in 0.1 M PBS with 0.3% Triton X-100 plus 5% NDS. Secondary antibodies were diluted in 0.1 M PBS with 0.3% Triton X-100 plus 1% NDS. Tissue was incubated overnight at 4°C in primary antibody, washed with 0.1 M PBS with 0.3% Triton X-100 plus 1% NDS, and then incubated for 2 hrs in secondary antibody at RT. Tissue was then washed with 0.1 M PB, and coverslipped with Fluoromount-G mounting medium (Southern Biotech). Images were acquired with a Leica TCS SPE confocal microscope. We used the following antibodies and reagents: sheep anti-CGRP (Abcam, 1:1,500); IB4 (Sigma-Aldrich, 1:1,000); guinea pig anti-Substance P (gift from J. Maggio, 1:10,000), Alexa Fluor 488 donkey anti-sheep; Alexa Fluor 555 streptavidin, Alexa Fluor 647 donkey anti-guinea pig.

### Statistical analysis

All data are displayed as the means +/- SEM unless otherwise noted. Dose–response data was fitted using a sigmoidal function with variable slope (Prism 5, GraphPad Software, San Diego, CA). Where more than 2 groups were employed, ANOVA analysis was applied with the Tukey test applied post-hoc. Pair-wise correlations and the corresponding p values were calculated using R (http://www.r-project.org). The p values were then adjusted using the Benjamini-Hochberg method to control for false-discovery rate [56].

## Endnote

^a^Zheng et al. Abcb5 Alleles Affect Susceptibility to Haloperidol-Induced Toxicity in Mice and Humans. Manuscript submitted.

## Electronic supplementary material

Additional file 1: Figure S1: Additional opioid adaptive traits in 23 strains of inbred mice. Panel **A**: Opioid-induced hyperalgesia (OIH) measured using thermal paw withdrawal (Hargreaves’) testing; Panel **B**: OIH measured using the tail flick assay; Panel **C**: Physical dependence measured using naloxone-precipitated jumping behavior; Panel **D**: Morphine tolerance measured as the shift in ED_50_ after 4 days of morphine treatment. For panels A and B, nociceptive thresholds after morphine treatment were divided by baseline values to obtain the fraction of baseline values. For each trait the mean value is displayed +/- S.E.M., n = 8 mice per strain. (JPEG 40 KB)

Additional file 2: Figure S2: The genomic positions as well as the amino acid residues that the SNPs change are shown in the first two rows. For each strain, possessing the reference (C57/6J) allele is coded as blue. Yellow signifies the alternative allele. Based on the resulting pattern, the 23 strains were divided into 3 groups: the strains in the first two groups have predominantly high fractional changes in mechanical nociceptive thresholds after morphine exposure (>0.5), while the strains in the 3rd group all have low fractional changes after morphine exposure (<0.5). (DOCX 15 KB)

Additional file 3: Table S1: Strain names and Jackson Laboratories catalog numbers for experimental mice. (DOCX 14 KB)
