# Supplementary figures and images for: The Netrin-1 receptor DCC is a regulator of maladaptive responses to chronic morphine administration
Source: BMC Genomics. 2014 May 8;15(1):345. doi: 10.1186/1471-2164-15-345 (PMC4038717; doi:10.1186/1471-2164-15-345)

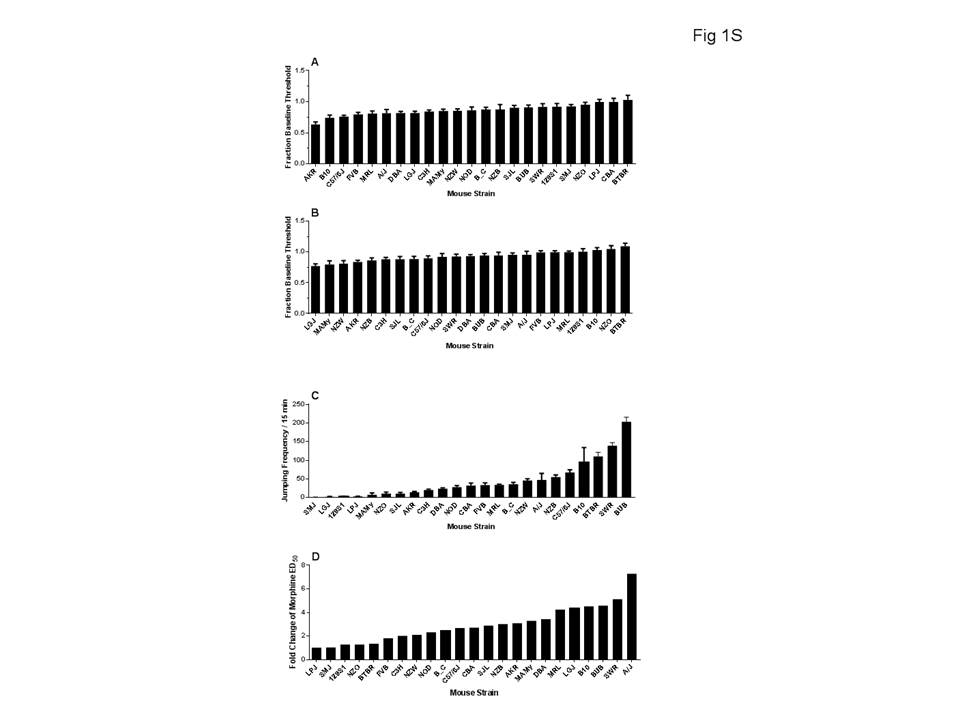

Supplement: Supplementary file 1 — Additional file 1: Figure S1: Additional opioid adaptive traits in 23 strains of inbred mice. Panel A: Opioid-induced hyperalgesia (OIH) measured using thermal paw withdrawal (Hargreaves’) testing; Panel B: OIH measured using the tail flick assay; Panel C: Physical dependence measured using naloxone-precipitated jumping behavior; Panel D: Morphine tolerance measured as the shift in ED50 after 4 days of morphine treatment. For panels A and B, nociceptive thresholds after morphine treatment were divided by baseline values to obtain the fraction of baseline values. For each trait the mean value is displayed +/- S.E.M., n = 8 mice per strain. (JPEG 40 KB) [file 12864_2013_6072_MOESM1_ESM.jpeg]
