# Supplementary material for: The Netrin-1 receptor DCC is a regulator of maladaptive responses to chronic morphine administration
Source: BMC Genomics. 2014 May 8;15(1):345. doi: 10.1186/1471-2164-15-345 (PMC4038717; doi:10.1186/1471-2164-15-345)
Supplement: Supplementary file 2 — Additional file 2: Figure S2: The genomic positions as well as the amino acid residues that the SNPs change are shown in the first two rows. For each strain, possessing the reference (C57/6J) allele is coded as blue. Yellow signifies the alternative allele. Based on the resulting pattern, the 23 strains were divided into 3 groups: the strains in the first two groups have predominantly high fractional changes in mechanical nociceptive thresholds after morphine exposure (>0.5), while the strains in the 3rd group all have low fractional changes after morphine exposure (<0.5). (DOCX 15 KB) [file 12864_2013_6072_MOESM2_ESM.docx]

**Figure S2.** The variation pattern of the two SNPs that induce amino acid changes in DCC

| Position | 71459153 | 71606017 |  | Mechanical threshold change |
| --- | --- | --- | --- | --- |
| Residue change | P1295S | A713V |  |  |
| A_J | 0 | 0 |  | HIGH |
| AKR | 0 | 0 |  | HIGH |
| B_C | 0 | 0 |  | HIGH |
| B10 | 0 | 0 |  | HIGH |
| BUB | 0 | 0 |  | HIGH |
| C57BL/6 J | 0 | 0 |  | HIGH |
| FVB | 0 | 0 |  | HIGH |
| LGJ | 0 | 0 |  | HIGH |
| MRL | 0 | 0 |  | HIGH |
| NOD | 0 | 0 |  | HIGH |
| NZB | 0 | 0 |  | HIGH |
| SMJ | 0 | 0 |  | LOW |
|  |  |  |  |  |
| C3H | 1 | 0 |  | HIGH |
| CBA | 1 | 0 |  | HIGH |
| DBA | 1 | 0 |  | HIGH |
| MAMy | 1 | 0 |  | HIGH |
| NZO | 1 | 0 |  | LOW |
| NZW | 1 | 0 |  | HIGH |
| SJL | 1 | 0 |  | HIGH |
| SWR | 1 | 0 |  | HIGH |
|  |  |  |  |  |
| 129S1 | 0 | 1 |  | LOW |
| BTBR | 0 | 1 |  | LOW |
| LPJ | 0 | 1 |  | LOW |
