# Supplementary material for: The Netrin-1 receptor DCC is a regulator of maladaptive responses to chronic morphine administration
Source: BMC Genomics. 2014 May 8;15(1):345. doi: 10.1186/1471-2164-15-345 (PMC4038717; doi:10.1186/1471-2164-15-345)
Supplement: Supplementary file 3 — Additional file 3: Table S1: Strain names and Jackson Laboratories catalog numbers for experimental mice. (DOCX 14 KB) [file 12864_2013_6072_MOESM3_ESM.docx]

**Table 1. Strain names and Jackson Laboratories catalog numbers for experimental mice.**

| **Strain Name** | **Jax Stock Number** |
| --- | --- |
| 129S1/SvImJ  A/J  AKR/J  B10.D2-H2/n2SnJ  Balb/cJ  BTBR T+ Itpr3tf/J  BUB/BnJ  C3H/HeJ  C57BL/6J  CBA/J  DBA/2J  FVB/NJ  LG/J  LP/J  MA/MyJ  MRL/MpJ  NOD/LtJ  NZB/BlnJ  NZO/HlLtJ  NZW/LacJ  SJL/J  SM/J  SWR/J | 2448  646  648  462  651  2282  653  659  664  656  671  1800  675  676  677  486  1976  684  2105  1058  686  687  689 |
|  |  |
